# 3D Biomimetic Scaffold for Growth Factor Controlled Delivery: An In-Vitro Study of Tenogenic Events on Wharton’s Jelly Mesenchymal Stem Cells

**DOI:** 10.3390/pharmaceutics13091448

**Published:** 2021-09-10

**Authors:** Maria Camilla Ciardulli, Joseph Lovecchio, Pasqualina Scala, Erwin Pavel Lamparelli, Tina Patricia Dale, Valentina Giudice, Emanuele Giordano, Carmine Selleri, Nicholas Robert Forsyth, Nicola Maffulli, Giovanna Della Porta

**Affiliations:** 1 Department of Medicine, Surgery and Dentistry, University of Salerno, Via S. Allende, 84081 Baronissi, Italy; mciardulli@unisa.it (M.C.C.); pscala@unisa.it (P.S.); elamparelli@unisa.it (E.P.L.); vgiudice@unisa.it (V.G.); cselleri@unisa.it (C.S.); nmaffulli@unisa.it (N.M.); 2 Department of Electrical, Electronic and Information Engineering “Guglielmo Marconi” (DEI), University of Bologna, Via dell’Università 50, 47522 Cesena, Italy; joseph.lovecchio@unibo.it (J.L.); emanuele.giordano@unibo.it (E.G.); 3 Guy Hilton Research Centre, School of Pharmacy and Bioengineering, Keele University, Stoke-on-Trent, Staffordshire ST4 7QB, UK; t.p.dale@keele.ac.uk (T.P.D.); n.r.forsyth@keele.ac.uk (N.R.F.); 4 Hematology and Transplant Center, University Hospital “San Giovanni di Dio e Ruggi D’Aragona”, 84131 Salerno, Italy; 5 Health Sciences and Technologies-Interdepartmental Center for Industrial Research (HST-ICIR), University of Bologna, Via Tolara di Sopra 41/E, 40064 Ozzano dell’Emilia, Italy; 6 Advanced Research Center on Electronic Systems (ARCES), University of Bologna, Via Vincenzo Toffano 2/2, 40125 Bologna, Italy; 7 Clinical Pharmacology, University Hospital “San Giovanni di Dio e Ruggi D’Aragona”, 84131 Salerno, Italy; 8 Centre for Sport and Exercise Medicine, Barts and The London School of Medicine, Queen Mary University of London, London E1 4NL, UK; 9 Research Centre for Biomaterials BIONAM, Università di Salerno, Via Giovanni Paolo II, 84084 Fisciano, Italy

**Keywords:** human Wharton’s Jelly Mesenchymal Stem Cells, hGDF-5 controlled delivery, PLGA nanocarriers, 3D fibrin scaffold, tenogenic commitment, cyclic strain bioreactor systems

## Abstract

The present work described a bio-functionalized 3D fibrous construct, as an interactive teno-inductive graft model to study tenogenic potential events of human mesenchymal stem cells collected from Wharton’s Jelly (hWJ-MSCs). The 3D-biomimetic and bioresorbable scaffold was functionalized with nanocarriers for the local controlled delivery of a teno-inductive factor, i.e., the human Growth Differentiation factor 5 (hGDF-5). Significant results in terms of gene expression were obtained. Namely, the up-regulation of Scleraxis (350-fold, *p* ≤ 0.05), type I Collagen (8-fold), Decorin (2.5-fold), and Tenascin-C (1.3-fold) was detected at day 14; on the other hand, when hGDF-5 was supplemented in the external medium only (in absence of nanocarriers), a limited effect on gene expression was evident. Teno-inductive environment also induced pro-inflammatory, (IL-6 (1.6-fold), TNF (45-fold, *p* ≤ 0.001), and IL-12A (1.4-fold)), and anti-inflammatory (IL-10 (120-fold) and TGF-β1 (1.8-fold)) cytokine expression upregulation at day 14. The presented 3D construct opens perspectives for the study of drug controlled delivery devices to promote teno-regenerative events.

## 1. Introduction

Tendon injuries generate pain, swelling, loss of function of the tendon itself and nearby structures, and instability. Conservative management involves physical therapy and pharmacological treatment with non-steroidal anti-inflammatory drugs (FANS), corticosteroids, narcotics, and viscosupplementation. Surgical procedures are elected when traditional modalities fail. Even if these approaches lead to a relatively high rate of success, they sometimes present limitations [1]. Tendon tissue poorly responds to current treatments, resulting in permanent changes of the native tendon structures (with scar tissue formation and fibrosis) and biomechanics. The inability of complete healing derives from the nature of the tendon itself. It is poorly cellularized and vascularized, and has a low metabolism [2,3].

In this context, in vitro models that allow the study of tenogenic events are important to improve pharmacological approaches and to develop advanced surgical devices. Human stem cells derived from bone marrow or adipose tissue aspirate are largely used for this purpose; whereas, those collected from cord blood and umbilical cord-derived Wharton’s Jelly have an increasing interest for tendon regenerative medicine studies. Stem cells can promote healing activity due to the production of cytokines, growth factors, and extracellular vesicles (such as exosomes), all involved in the regeneration processes [4,5,6]. On the other hand, tissue engineering (TE) approaches involving biopolymers and bioreactors can recreate biomimetic environments with specific microarchitectural and biomechanical inputs to properly stimulate cells toward a specific phenotype, promoting an improved understanding of tendon biology and related regenerative processes [7].

Among the different types of stem cells adopted in TE approaches for tendon regeneration studies [8], mesenchymal stem cells from the Wharton’s Jelly of the human umbilical cord (hWJ-MSCs) have potential in the future of regenerative medicine and tenogenesis studies [9,10]. Wharton’s Jelly is a connective tissue of the umbilical cord located between the umbilical vessels and the amniotic epithelium. This gelatinous substance has an extracellular matrix (ECM) containing collagen, hyaluronic acid, sulfated proteoglycans, growth factors, cytokines, extracellular vesicles, and primitive mesenchymal stem cells [11]. Compared to bone marrow- and adipose-derived collection procedures, the ease of harvest of hWJ-MSCs does not pose donor site morbidity where every birth represents an opportunity to collect materials for research and clinical applications [12]. hWJ-MSCs resemble embryonic stem cells and have attractive expansive properties and immunomodulatory characteristics [9,13]. hWJ-MSCs are able to differentiate into tenogenic lineages in response to signal transduction mediated by human Growth Differentiation Factor-5 (hGDF-5) [13], a well-known growth factor belonging to the Transforming Growth Factor-β superfamily capable of triggering the expression of genes linked to the neotendon phenotype [14,15,16,17,18].

Growth factors play a predicted role in tendon development and repair and are secreted by a variety of cells, such as tendon progenitor cells, epithelial and vascular endothelial cells, fibroblasts, and inflammatory cells. Following tissue damage, growth factors are released and bind to membrane receptors and activate intracellular signaling pathways involved in the transcriptional expression of genes linked to proliferation, differentiation, and matrix synthesis, influencing the healing process [19]. hGDF-5 seems to be involved in cytoskeleton reorganization, cell adhesion, and ECM remodeling during tenogenic differentiation [20].

Furthermore, the tendon is a mechanosensitive tissue and ECM remodeling is influenced by mechanical stimulation [21,22]: prolonged rehabilitation is considered an efficient alternative to surgical procedures and pharmacological therapy [23]. Tendon homeostasis, development, and healing are driven by applied mechanical forces; mechanotransduction processes translate mechanical loads into biochemical signals linked to key signaling pathways in tendon cells [24,25,26]. Mechanical stimulation has been delivered to stem cells in tissue engineering approaches to promote tenogenic differentiation and matrix organization; specifically, strain has a key role in tenogenic differentiation induction [27,28,29].

A biomimetic environment can be achieved by merging three-dimensional (3D) scaffolds and bioreactors to transfer biochemical stimuli and mechanical loads to cells. Scaffolds replicate the ECM by supporting cell growth and differentiation while being bioresorbable and supporting mechanical loads [30,31,32,33,34]. Hydrogels are biocompatible but, to overcome their poor mechanical properties, they can require combination with more force-resistant biopolymers [29,35]. Several bioreactors have been used to impart, in a controlled manner, mechanical forces to cells in culture, including tenogenic mechanical stimuli [29,35,36,37,38,39,40,41,42].

Controlled delivery of biochemical stimuli, such as human growth factors, is still a challenge in TE protocols, but it is necessary to overcome the limits associated with standard culture medium supplementation [43]. Poly-lactic acid (PLA) and poly-lactic-co-glycolic acid (PLGA) carriers (FDA-approved bioresorbable polymers) have been recently applied. These carriers can act as micro-environmental regulators within a 3D bioengineered scaffold, providing a spatio-temporally controlled delivery of biomolecules [43,44], in both pharmaceutical [45,46,47] and biomedical [48,49,50] fields.

Here, we used a previously described bioengineered scaffold with tenoinductive potential [29] to study the tenogenic commitment of hWJ-MSCs. The 3D scaffold featured a braided hyaluronate elastic band and a fibrin hydrogel, capable of carrying poly-lactic-co-glycolic acid nano-carriers (PLGA-NCs) loaded with hGDF-5; these nanosystems were fabricated by a proprietary technology to assure the proper release profile. To understand the effect of hGDF-5 sustained delivery within the 3D fibrin hydrogel, a series of controlled experiments were performed. In all experiments, the braided band underwent a specific cyclic strain along 14 days of culture through a custom-made bioreactor that ensured mass transfer within the culture system. The selection of hWJ-MSCs was preferred to also investigate their potential use as in vitro model for tendon regeneration and gene expression of type I Collagen, Scleraxis-A, Decorin, Tenascin-C, and type III Collagen was evaluated to monitor cell tenogenic commitment. Picro-Sirius Red staining was used to highlight collagen deposition and cell interaction with the synthetic ECM. Moreover, the immunomodulatory properties of hWJ-MSCs along commitment events were explored: the expression of pro-inflammatory (IL-6, TNF, IL-12A, IL-1β) and anti-inflammatory (IL-10, TGF-β1) cytokines was thus investigated under the best tenoinductive conditions.

## 2. Materials and Methods

### 2.1. hWJ-MSCs

Human Wharton’s Jelly Mesenchymal Stem Cells (hWJ-MSCs) were obtained from two donors (aged 23, 31) who gave written informed consent to use their umbilical cord for research purposes, in compliance with the Declaration of Helsinki. The protocol was approved by Our Institutional Review Board (Ethic Committee “Campania Sud”, Brusciano, Naples, Italy; prot./SCCE n. 24988). Further indication on hWJ-MSCs isolation and harvesting, flow cytometry, and gating strategy are described in Supplementary Materials (text and figure).

### 2.2. PLGA-NCs Characterization and hGDF-5 Release Profile

PLGA nano-carriers (PLGA-NCs) were produced using Supercritical Emulsion Extraction (SEE) technology, which enables rapid polymer NCs production starting from multiple emulsions. The oily phase organic solvent is extracted via dense gas utilizing a countercurrent packed tower operating in continuous mode [51]. In detail, recombinant hGDF-5 (PeproTech, London, UK) was solubilized with 0.1% (*w*/*v*) human serum albumin (HSA; Sigma-Aldrich, Milan, Italy) plus 0.06% polyvinyl alcohol (PVA). HSA was included in the water internal phase as a growth factor stabilizer. This solution was added to the oily phase composed of 500 mg of PLGA (RG 502H, 7000–17,000 kDa, Evonik, Essen, Germany) in 5 mL of Ethyl Acetate (EA, purity 99.9%). The primary emulsion was then slowly added into EA-saturated aqueous Tween 80 solution (supplemented with 15% *w*/*v* of glucose) by high-speed stirring (mod. L4RT, Silverson Machines Ltd., Waterside, Chesham, Bucks, UK). All emulsions were processed immediately following preparation. SEE technology operative conditions were set at 8 MPa and 38 °C in the high-pressure column, with a Carbon Dioxide (CO_2_) flow of 1.4 kg/h and a Liquid/Gas ratio of 0.1 (*w*/*w*) [52]. Carrier suspension was recovered at the bottom of the extraction column and then was washed and lyophilized; 70% of the loaded biopolymer was recovered at the end of each run. Carrier particle size distributions (PSDs) were analyzed using a laser granulometer (mod. Mastersizer S; Malvern Instruments Ltd., Worcestershire, UK), proceeding from dynamic light scattering (DLS). Sizes are expressed in nanometers (nm) as volume mean size (MS), with standard deviation (SD). The morphology and shape of the carriers were investigated using a field emission-scanning electron microscopy (FE-SEM; mod. LEO 1525; Carl Zeiss, Oberkochen, Germany). Samples were glued on an aluminum stub covered by a double-sided adhesive carbon tape and coated with a gold film (250 A thickness) by means of a sputter coater (mod.108 A; Agar Scientific, Stansted, UK). hGDF-5 release profile was monitored in vitro suspending 5 (±0.3) mg of NCs in 0.5 mL of PBS 1X plus 0.1% *w*/*w* Tween 20. Samples were placed in incubator at fixed temperature (37 °C), and stirred continuously at 100 rpm. Every 24 h, they were centrifuged (14,000 rcf, 10 min) and the supernatant was collected and replaced with fresh media to maintain sink conditions. Released hGDF-5 concentrations from each sample were analyzed using an Enzyme Linked Immunosorbent Assay (ELISA, Cloud-Clone Corp., Houston, TX, USA). Release experiments were performed in duplicate (*n* = 2); the curve describes the mean profile as ng/g (protein released/PLGA-NCs) versus time.

### 2.3. Scaffold Preparation and Characterization

The elastic modulus of the hyaluronate band alone or embedded within the 3D fibrin hydrogel was measured according to the ASTM 1708 by a CMT 6000 dynamometer (SANS, Shenzhen, China) equipped with a 100 N load cell. Samples were shaped to obtain specimens having a gauge length (Lo) of 22 mm and a width (W) of 5 mm; sample thickness (S) was 0.5 mm. A monoaxial deformation was applied to the sample with a speed of 22 mm/min, and force (F) and elongation (L) during traction was registered. The value of force (F) provided by the instrument was divided by the sample area (A = W × S) to obtain the strength values (σ = F/A). The deformation values (L) during the run were compared to the initial length to obtain values of strain (ε = (L − Lo)/Lo); the ultimate tensile strength (σ_max_, expressed in MPa) was calculated as load to failure/cross sectional area of the sample.

For each 3D scaffold, a combination of 50 mg/mL fibrinogen from human plasma (Sigma-Aldrich, Milan, Italy), 15,600 U/mL aprotinin (Sigma-Aldrich, Milan, Italy), and α-MEM (Corning, NY, USA) supplemented with 10% FBS, was added with a 1:1:1 ratio to an average of 1 × 10^6^ cells/mL and 80 mg of PLGA-NCs (hGDF-5 loading: 3 μg/g). A homogeneous cells/PLGA-NCs/fibrinogen suspension was then dripped into a mold (30 × 20 × 4.5 mm) containing the braided band; free ends were left to enable scaffold fixation into the culture chamber of the bioreactor. Upon addition of 100 U/mL thrombin (Sigma-Aldrich, Milan, Italy), fibrin polymerization was allowed, placing the mold in a 37 °C humidified incubator for 30 min. After the incubation time, a uniformly distributed hydrogel was formed and the band was entrapped inside it.

The scaffold was then transferred from the mold to the bioreactor culture chamber, containing 20 mL culture media (α-MEM plus 10% FBS), and placed in an incubator (37 °C, 5% CO_2_ atmosphere and 95% relative humidity). For the histochemical analysis, at different time points, a section of the scaffold was fixed in 4% PFA at 4 °C for 4 h, washed in PBS 1X (RT, 10 min, 3 times), incubated in 30% sucrose overnight to allow cryo-protection, included in OCT embedding medium, and then frozen at −20 °C for cryostat sectioning (slices of 10 μm of thickness). The remaining portion of the scaffold was lysed in QIAzol^®^ Reagent for total RNA extraction.

### 2.4. Cyclic Strain Bioreactor Description and Cytotoxicity Study

A custom-made bioreactor system was used to apply a cyclic deformation to the 3D scaffold within an ad hoc designed 20 mL culture chamber. The sample was clamped at its free ends between a motionless stand and a sliding arm. This latter was connected to a rod driven by a linear motor (mod. 42BYGH48; 1.8, 1.2 A, 0.4 Nm, DFA) actuating the desired deformation protocol. All the components of this system were manufactured with a Form3 printer (Formlabs, Somerville, MA, USA) using a biocompatible Dental Clear LT^®^ resin. A dedicated graphical user interface allowed for the tuning of the system operations. The scaffold was deformed (40 h of stretching followed by 6 h of rest), providing 10% of elongation of the initial (30 mm) total scaffold length at a frequency of 1 Hz.

The biocompatibility of the Dental Clear LT^®^ 3D-printed components was tested using CHO-K1 (P5) and HeLa (P14) cell lines. Cells were seeded on coverslips in 24-well plates at a density of 30,000 cells/well; after 24 h, the coverslips were transferred in the culture chamber of the bioreactor or in new standard well plates (control), both containing Dulbecco’s Modified Eagle’s Medium (DMEM) supplemented with 10% FBS (Corning Cellgro, Manassas, VA, USA), 1% Glutagro^TM^ (Corning Cellgro, Manassas, VA, USA), and 1% Penicillin-Streptomycin solution. Cytotoxicity was evaluated after 24 h and 48 h using MTT assay. Then, 500 μL of MTT was added (1 mg/mL final concentration) to each well, containing cells seeded on coverslips, and incubated at 37 °C for 4 h, protecting the plate from the light. Formazan salts were dissolved in 500 μL of DMSO. The experiments were performed in triplicate for each time point. The absorbance was measured at 490 nm with UVvis system Tecan (mod. Infinite-M200 Pro). Cell viability was calculated as the percentage of the control group, considered as 100%. The percentage viability of cells was calculated according to Equation (1):

(1) $$\%\;\mathrm{Cell}\;\mathrm{viability}=\frac{\mathrm{Abs}\;\mathrm{of}\;\mathrm{sample}\;-\;\mathrm{Abs}\;\mathrm{blank}}{\mathrm{Abs}\;\mathrm{of}\;\mathrm{control}\;-\;\mathrm{Abs}\;\mathrm{of}\;\mathrm{blank})\;}\times 100$$

For the cytotoxicity investigation on cells within the 3D scaffold, the bioengineered construct was assembled, as described above, adding HeLa cells to the fibrin hydrogel. A cell density of about 1 × 10^6^/mL (P14) was used. The scaffold was placed in the culture chamber of the bioreactor, containing 20 mL DMEM supplemented with 10% FBS (Corning Cellgro, Manassas, VA, USA), 1% Glutagro^TM^ (Corning Cellgro, Manassas, VA, USA), and 1% Penicillin-Streptomycin solution. The viability of cells into scaffolds was detected by Live/Dead assay (Calcein AM solution 4 μM and Ethidium homodimer I solution 2 μM, Sigma-Aldrich, Milan, Italy), after 24 h and 72 h. Cells were stained for 1 h at 37 °C, washed in PBS 1X and imaged using a fluorescence microscope (mod. Eclipse, Nikon, DE). Green emission of the Calcein dye stains the cytosol of live cells, while red emission of cell membrane-impermeable ethidium homodimer-1 dye stains nuclei of dead cells. However, the braided band fibers retained red dye, preventing accurate quantification of the red channel. Consequently, only the green signal given by live cells was quantified.

Signal quantification was performed on images in a blinded manner using ImageJ analysis software (National Institutes of Health, Bethesda, MD, USA) measuring the pixel intensity of green areas where live cells were present [53,54]. Original images were first converted into a gray scale (16-bit) from RGB format. Then, the average value of pixel intensity ranging from 0 (dark) to 255 (white) was calculated for the single images. A minimum of 10 fields (images) were used for the analysis for each experiment at each time point. Data were expressed as fold change over T0 = 1.

### 2.5. RNA Isolation and Gene Expression Profiles by Quantitative Reverse Transcription PCR (RT-qPCR)

Total RNA was extracted from hWJ-MSCs seeded into each 3D scaffold using QIAzol^®^ Lysis Reagent (Qiagen, Hilden, Germany), chloroform (Sigma-Aldrich, Milan, Italy), and the RNeasy Mini Kit (Qiagen, Hilden, Germany). Then, the iScriptTM cDNA synthesis kit (Bio-Rad, Milan, Italy) was used to reverse-transcribe 300 ng of total RNA for each sample. Relative gene expression analysis was performed in a LightCycler^®^ 480 Instrument (Roche, Milan, Italy), using the SsoAdvanced^TM^ Universal SYBR^®^ Green Supermix (Bio-Rad) and the validated primers for SCX-A, DCN, COL1A1, TNC, COL3A1, IL-6, TNF, IL-12A, IL-1β, IL-10, and TGF-β1 (Bio-Rad), according to MIQE guidelines [55]. Triplicate experiments were performed for each condition studied and data were normalized to GAPDH expression. The geNorm method [56] was applied to calculate reference gene stability between the different conditions (calculated with CFX Manager software (Version 3.1, Bio-Rad Laboratories, Milan, Italy); M < 0.5). Fold changes were determined using the 2^−ΔΔCp^ method, and presented as relative levels over T0 = 1.

### 2.6. Immunohistochemical Assay

The Picro-Sirius Red Stain Kit (Polysciences, Inc., Warrington, PA, USA) was used to perform the Sirius Red staining. Sections with a thickness of 10 μm were: stained in hematoxylin for 8 min, washed in water for 2 min, immersed into phosphomolybdic acid for 2 min, washed in water for 2 min, dipped into Picrosirius Red F3BA Stain for 60 min, and then into HCl 0.1 M solution for 2 min. The sections were dehydrated in solutions at increasing ethanol gradient (70%–75%–95%–100%) and finally immersed into xylene for 5 min. Samples were mounted using Eukitt medium and dried under the chemical hood for 30 min.

### 2.7. Statistical Analysis

GraphPad Prism software (Version 6.0 for Windows, GraphPad Software, Inc., San Diego, CA, USA) were used for statistical analysis of data obtained from multiple experiments, expressed as mean ± SD. The statistical significance was analyzed using ANOVA test for independent groups; differences were considered statistically significant when *p* ≤ 0.05 [57].

## 3. Results

### 3.1. Cyclic Strain Bioreactor Cytotoxicity

**Figure 1:**
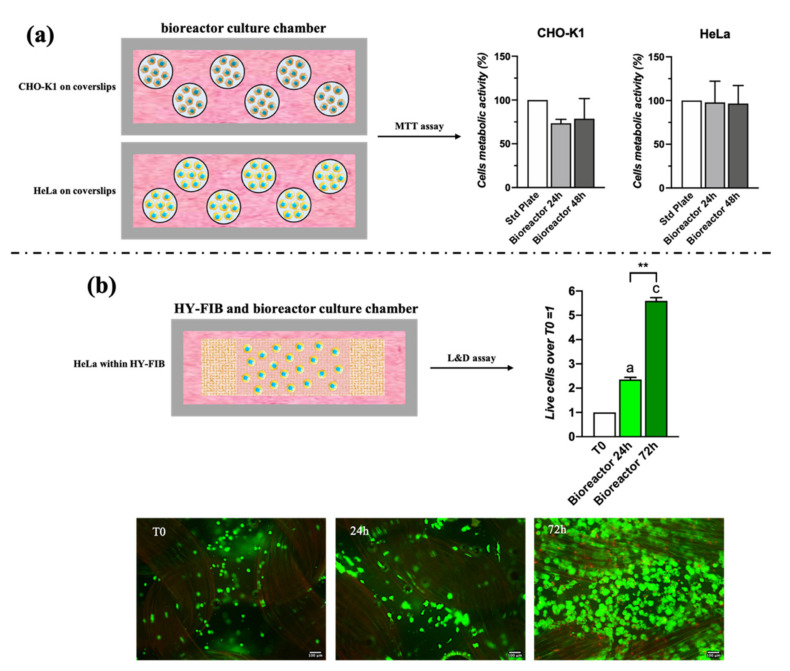
Cytotoxicity assay of 3D printed cyclic strain bioreactor with CHO-K1 and HeLa cells. MTT assay on CHO-K1 and HeLa cells seeded on coverslips at 24 h and 48 h of culture in the bioreactor chamber. The histograms report the mean percentage of viable cells compared to control (cells cultured in a standard plate, 100%) (**a**). Live and Dead assay at 24 h and 72 h on HeLa cells embedded in the 3D fibrin hydrogel of the 3D scaffold. The green signal, indicating viable cells, was quantified using ImageJ software and presented as fold change over T0 = 1 (**b**). Statistically significant differences are shown as ** = *p* ≤ 0.01 compared to T0. Scale bar = 100 μm.

The cyclic strain bioreactor was specifically designed and 3D printed; therefore, before its use with human stem cells, its cytotoxicity was evaluated using CHO-K1 and HeLa cell lines. The study revealed that bioreactor vessel and elements did not affect cell metabolic activity, which was 80% for CHO-K1 cells and 100% for HeLa cells at 24 h and 48 h (Figure 1a). Cytotoxicity was also evaluated on the 3D scaffold bioengineered with 1 × 10^6^ HeLa and maintained for 72 h in the bioreactor chamber under cyclic strain, set at 10% deformation and 1 Hz frequency. Live and Dead assay indicated cells proliferation with an increased green signal (live cells) of 2-fold after 24 h and 5.5-fold after 72 h of culture (Figure 1b).

### 3.2. Scaffold Assembly with hWJ-MSCs and Its Mechanical Characterization

Flow cytometry characterization of hWJ-MSCs with data acquisition profiles is reported in Figure S1 (see Supplementary Materials). Cells were positive for CD90, CD105, and CD73, and negative for CD14, CD34, CD45, and HLA-DR according to previously published data [58].

**Figure 2:**
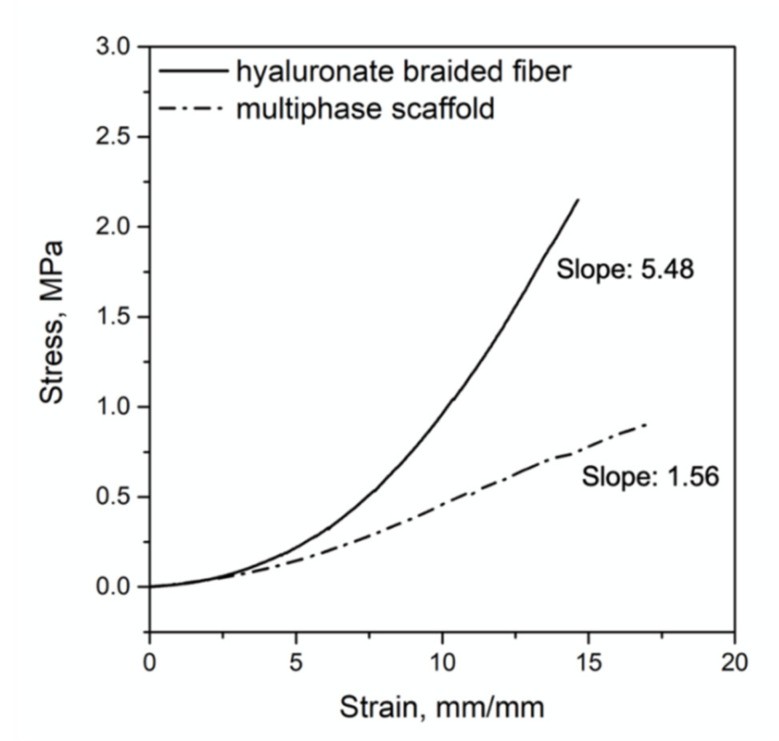
Mechanical characterization of the 3D scaffold. Stress–strain plot and elastic modulus values of hyaluronate braided band (continuous line) and of multiphase stem cell-based scaffold (dashed line).

Each 3D scaffold was assembled with 8 × 10^5^ hWJ-MSCs distributed within the fibrin hydrogel and a crosslinked hyaluronate band using a specific mold (30 × 20 mm; height: 3 mm), as indicated in the methods section. The braided hyaluronate band ensured the mechanical behavior of the overall scaffold and, coupled with the bioreactor, ensured strain delivery in a dynamic culture environment. Braided band alone had a tensile strength at break point of 3 MPa and a Young Modulus of 6 MPa. When the same measure was performed on the 3D bioengineered construct, the presence of the fibrin environment reduced the tensile strength at break point of about one third (1 MPa) as well as the Young modulus of the 3D system, which was measured as 2 MPa, as indicated by the data reported in Table 1 and Figure 2.

**Figure 3:**
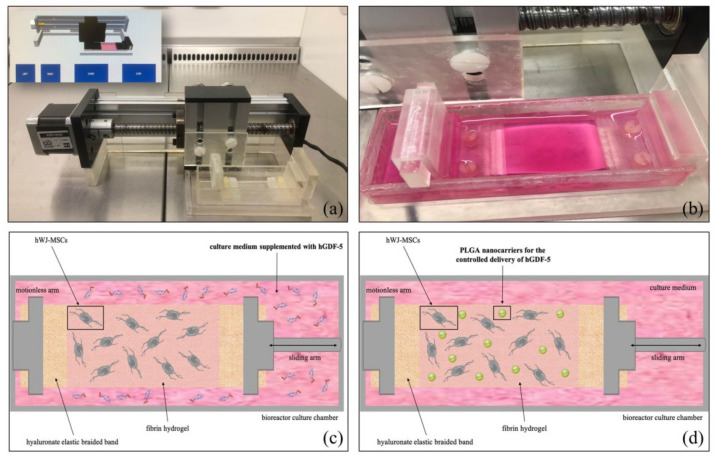
Cyclic strain bioreactor and of the 3D scaffold and representation of the two different experiments set up. Image of the cyclic strain bioreactor and software interface (**a**). 3D scaffold placed in the bioreactor culture chamber (**b**). Schematic representation of the two series of experiments performed: hGDF-5 was supplemented in the culture medium (**c**) or encapsulated within PLGA nanocarriers for its local controlled delivery (**d**).

The mechanical behavior of the scaffold was adequate to deliver a cyclic deformation of 10% with 1 Hz of frequency, as applied by the software control interface of the bioreactor (Figure 3a); the braided band of the scaffold was held at both free ends by a motionless arm and a sliding one, placed in the bioreactor chamber full of culture medium (Figure 3b) and exposed to the mechanical stimulation for 40 h (followed by 6 h of rest). In these conditions, the braided band provided not only a mean force distribution of 9 × 10^−5^ MPa, as calculated in a previous work [29], but at the same time assured convective mass transport of hGDF-5 within the 3D system.

Under this strain force, two series of experiments were performed: (i) supplementing hGDF-5 in the external medium (Figure 3c) at 100 ng/mL and changing the medium every 4 days; and (ii) adding, into the fibrin hydrogel, PLGA carriers providing a controlled release of the growth factor within the 3D system (Figure 3d).

**Figure 4:**
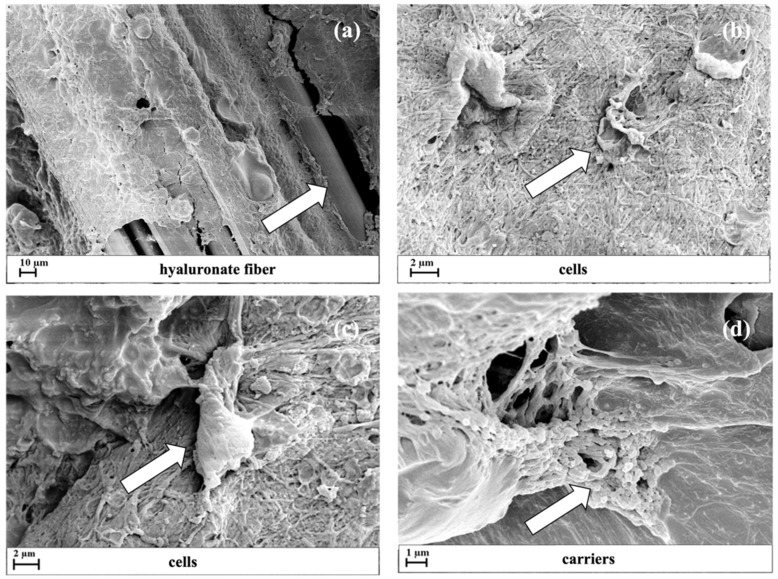
3D scaffold structure monitored by FE-SEM images after its assembly. FE-SEM images were reported with different enlargements to better describe: the hyaluronate fibers covered by the fibrin hydrogel (**a**); the cells (see arrowheads) entrapped within the fibrin matrix in large aggregates connected with fibrin network (**b**,**c**); NCs distributed within the fibrin network (**d**).

In the first series of runs, 100 ng/mL of hGDF-5 was supplemented in the external medium, as previously optimized [29,47]. When carriers were loaded within the system, 80 mg was added in each 3D assembled system in order to ensure similar growth factor concentrations within the 3D system through sustained release. In this last case, the multilevel scaffold structure was investigated by Field Emission Scanning Electron Microscopy (FE-SEM) where images displayed braided fibers with a mean diameter of 10 μm, uniformly covered by fibrin hydrogel (Figure 4a). From the images are also evident the cells immobilized within fibrin (Figure 4b,c) and NCs distributed within the same fibrin network (Figure 4d).

### 3.3. Bioengineered Scaffold in Dynamic Culture and hGDF-5 in the External Medium

**Figure 5:**
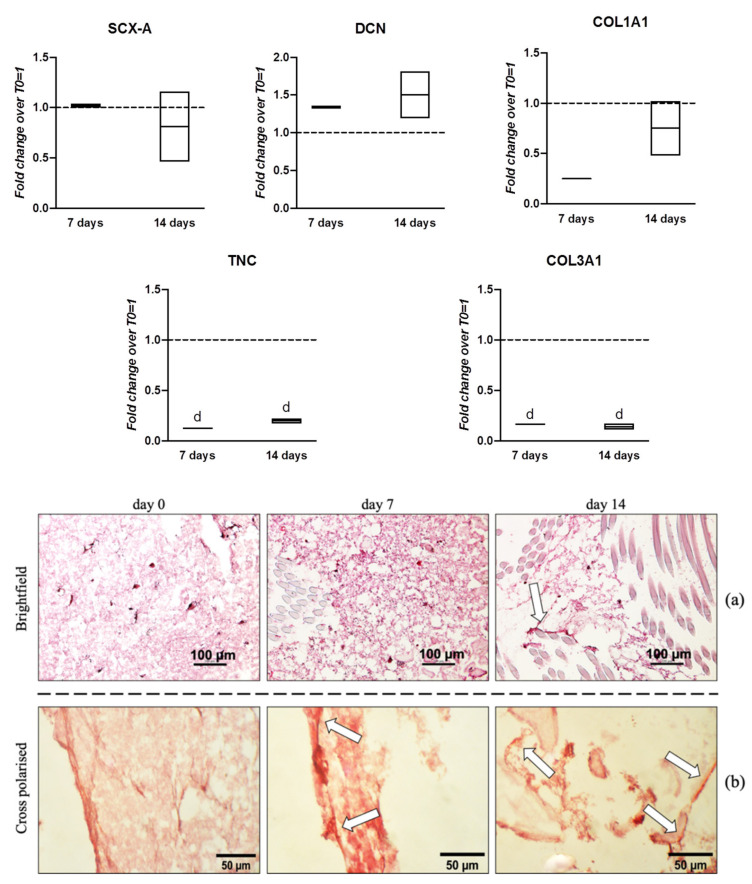
Gene expression profiles and histological characterization of hWJ-MSCs cultured within the 3D construct into a medium supplemented with hGDF-5 with cyclic strain. hWJ-MSCs were cultured up to 14 days. The mRNA levels of different tenogenic markers (COL1A1, SCX-A, DCN, TNC, and COL3A1) were monitored. Relative quantification of each mRNA gene expression normalized to endogenous GAPDH (internal control) was calculated using the 2^−ΔΔCt^ method and presented as fold change over hWJ-MSCs T0 = 1 (dashed line). Statistically significant differences are shown as “d” = *p* ≤ 0.001 compared to T0; *n* = 2 (biological replicates). Samples at same time-points were subjected to Sirius Red staining for collagen highlighting. The 3D fibrin matrix showed small areas stained in darker red probably filled with collagen (arrowheads, **a**). Polarized microscope revealed few birefringent collagen fibers (arrowheads, **b**).

In the first experimental setting, hWJ-MSCs were seeded within the fibrin hydrogel of the scaffold and cultured, under dynamic conditions, in a medium supplemented with 100 ng/mL of hGDF-5 for up to 14 days. Samples were collected after 7 days and 14 days to monitor tenogenic marker expression. In these conditions, DCN displayed a slight and constant up-regulation of 1.4-fold at Day 7 and 1.5-fold at Day 14 (Figure 5). Histological characterization was obtained by staining with Sirius Red for collagen highlighting at Days 7 and 14 of culture (Figure 5a,b). A homogenous network of synthetic fibrin matrix at Day 0 was observed with cells internally immobilized.

The 3D fibrin matrix maintained its integrity during the culture, even though it showed small areas stained in darker red (arrowheads, Figure 5a) potentially filled with collagen especially at day 14. Polarized microscopy revealed birefringent collagen fibers at day 14 (Figure 5b).

### 3.4. Bioengineered Scaffold in Dynamic Culture Loaded with PLGA/hGDF-5 Nanocarriers

**Figure 6:**
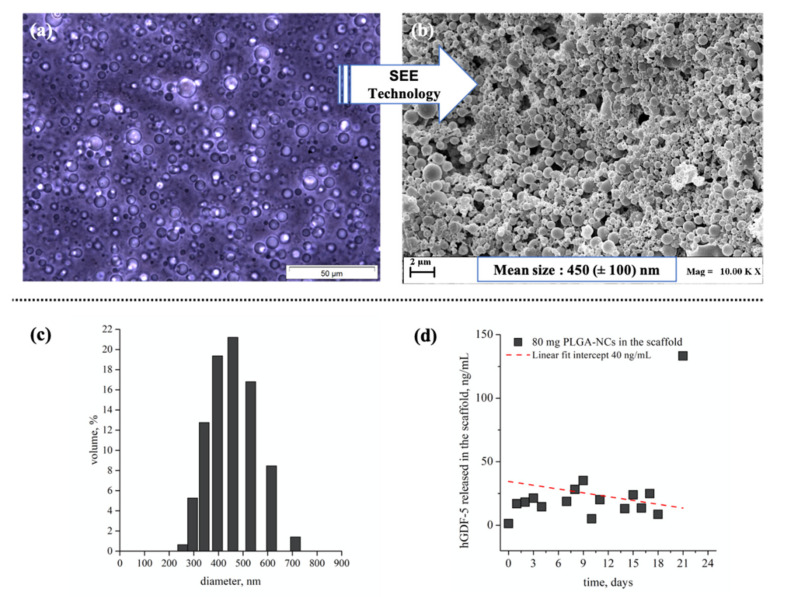
Images of emulsion and derived PLGA-NCs obtained by SEE technology, particle size distribution, and hGDF-5 release profiles within the 3D environment. Optical microscope image of emulsion (**a**) and electronic microscope image (**b**) of carriers obtained after emulsion processing by SEE technology; size distribution data of PLGA carriers expressed as volume percentage (**c**); in vitro hGDF-5 release profile (ng/mL/day) monitored at 37 °C and 100 rpm by ELISA-based assay from 80 mg of carriers, as loaded in each construct (**d**); *n* = 2.

Given these results, we assembled a 3D system with an anisotropic nano-to-macro architecture to observe if this configuration may enhance hWJ-MSCs tenogenic commitment. Indeed, functionalizing the fibrin hydrogel with polylactic-co-glycolic acid nanocarriers (PLGA-NCs) carrying human Growth Differentiation factor 5 (hGDF-5), and able to ensure a sustained delivery of the biochemical factor within the 3D scaffold, delivered an enhanced cell commitment. hGDF-5 loaded PLGA-NCs were obtained using Supercritical Emulsion Extraction technology, as described elsewhere [59]; carriers exhibited a spherical morphology with a mean size of 450 (±100) nm (Figure 6a–c) and a hGDF-5 loading of 3 μg/g, providing a daily release growth factor concentration of about 40 ng/mL within the 3D microenvironment, when 80 mg of carriers were incorporated in the hydrogel component of the 3D system (Figure 6d).

**Figure 7:**
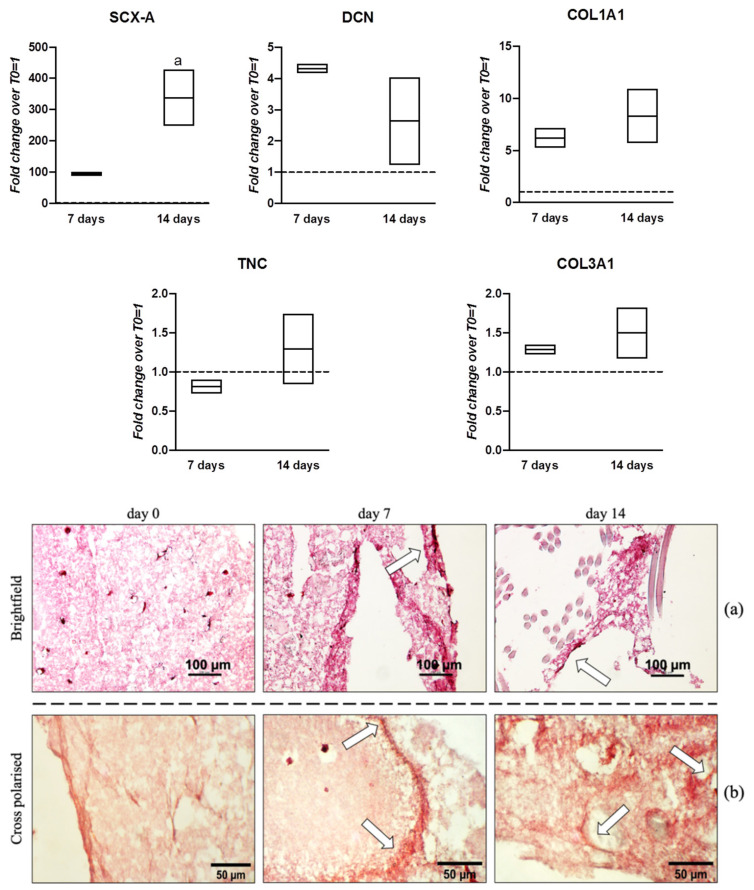
Gene expression profiles for tenogenic markers and histological characterization of hWJ-MSCs cultured within 3D construct functionalized with PLGA-NCs for hGDF-5 controlled delivery under cyclic strain. hWJ-MSCs were cultured into the 3D microenvironment for up to 14 days. The mRNA levels of different tenogenic markers (COL1A1, SCX-A, DCN, TNC, and COL3A1) were monitored. Relative quantification of each mRNA gene expression normalized to endogenous GAPDH (internal control) was calculated using the 2^−ΔΔCt^ method and presented as fold change over hWJ-MSCs T0 = 1 (dashed line). Statistically significant differences are shown as “a” = *p* ≤ 0.05 compared to T0; *n* = 2 (biological replicates). Samples at same time-points were subjected to Sirius Red staining for collagen highlighting. The homogenous network of synthetic fibrin matrix appeared progressively filled with new areas of matrix stained in darker red suggesting collagen deposition (arrowheads, **a**). Polarized microscope revealed larger areas of birefringent collagen fibers (arrowheads, **b**).

Cells cultured under dynamic conditions and with time-points at day 7 and day 14 were chosen to monitor the gene expression of tenogenic markers. COL1A1 levels displayed a 7-fold overexpression at day 7, rising slightly at day 14 (8-fold). SCX-A levels were substantially elevated at day 7 (100-fold), while an even stronger and significant increase (350-fold) was observed at day 14. On the contrary, DCN displayed up-regulation of 4.5-fold at day 7 before dropping to 2.5-fold at day 14. TNC did not show significant up-regulation, exhibiting expression levels close to T0. COL3A1 maintained a very slight up-regulation of 1.2-fold at day 7 and 1.5-fold at day 14 (Figure 7).

Collagen protein deposition within the 3D matrix was confirmed by Sirius Red staining (Figure 7a,b). The homogenous network of synthetic fibrin matrix with cells (observed at Day 0), appeared progressively filled with new areas of matrix that stained in darker red (arrowheads), suggesting collagen deposition within the matrix. Moreover, the overall matrix seemed contracted and was clearly rearranged over time (Figure 7a); further matrix characterization with polarized microscopy revealed large areas of birefringent collagen fibers at days 7 and 14 of culture (Figure 7b).

**Figure 8:**
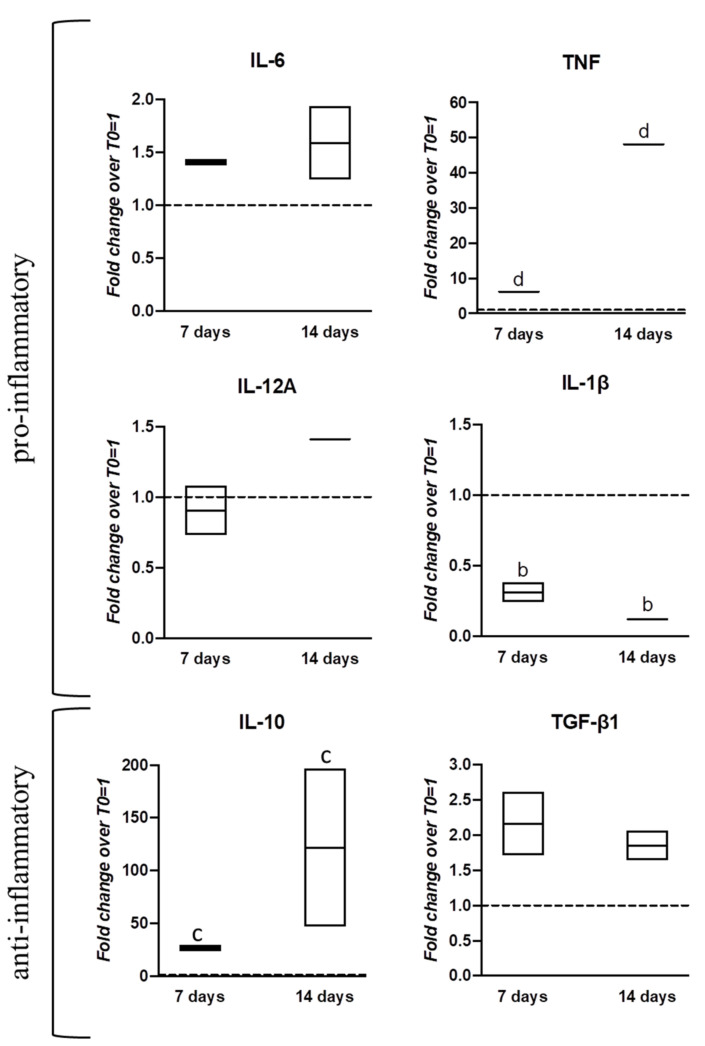
Gene expression profiles for cytokines by hWJ-MSCs cultured within scaffold assembled with PLGA-NCs for hGDF-5 controlled delivery. The mRNA levels of different pro-inflammatory (IL-6, TNF, IL-12A, IL-1β) and anti-inflammatory (IL-10, TGF-β1) cytokines were monitored. Relative quantification of each mRNA gene expression normalized to endogenous GAPDH (internal control) was calculated using the 2^−ΔΔCt^ method and presented as fold change over hWJ-MSCs T0 = 1 (dashed line). Statistically significant differences are shown as “b” = *p* ≤ 0.001; “c” = *p* ≤ 0.005; “d” = *p* ≤ 0.001; compared to T0; *n* = 2 (biological replicates). The overexpression of pro-inflammatory factors (IL-6, TNF) was significant, together with the upregulation of anti-inflammatory ones (IL-10, TGFβ1).

We finally evaluated cytokine transcript expression in the optimal tenoinductive setting: nanocarriers for hGDF-5 controlled release. The expression levels of IL-6 showed a slight and constant up-regulation (1.5-fold) across the time-points studied. At day 7, TNF showed a 5-fold increase, followed by a strong and significant upregulation of 45-fold at day 14. IL-12A showed no change (day 7) and a slight overexpression of 1.4-fold at day 14, while IL-1β remained significantly downregulated for the entire duration of the experiment. IL-10 exhibited a consistent profile of upregulation with 30-fold and 120-fold increases at day 7 and day 14, respectively; whereas TGF-β1 upregulation was slighter (2-fold) across the culture (Figure 8).

## 4. Discussion

A 3D biomimetic construct (composed of a hyaluronate elastic band covered by a fibrin hydrogel) was used for both cytotoxicity assay (using HeLa cell line) and to study the tenogenic commitment of human mesenchymal stem cells collected from Wharton’s Jelly. Cytoxicity data confirmed the safety of printed bioreactor elements for cell culture; whereas, mechanical characterization of the 3D system indicated that the braided band provided a mean force distribution of 9 × 10^−5^ MPa to the cells loaded within [29]. Despite several works reporting the concentration of 100 ng/mL as optimal for induction of stem cell commitment toward a tenogenic phenotype, when it was supplemented in the culture medium (wherein the 3D construct was immersed), we did not observe tenogenic gene up-regulation, probably because these indications mainly refer to monolayer cultures in conventional flasks [13,15,16]. Furthermore, hGDF-5 was already reported to commit WJ-MSCs towards a tenogenic phenotype [13] and the mechanical input provided was expected to improve the overall growth factor mass transfer within the 3D system [32], allowing its active transport through the cells loaded within the 3D scaffold [50]. However, in our case, a subsequent commitment was not observed in the above-described culture conditions.

The poor over-expression of tenogenic markers by hWJ-MSCs is also in contrast with our previous work, in which only mechanical force distribution, provided by the cyclic strain of the braided band, triggered the tenogenic commitment of human bone marrow mesenchymal stem cells (hBM-MSCs), even in the absence of specific growth factors [29]. However, no indication of hWJ-MSCs sensitiveness to mechanical inputs has ever been described in the literature; therefore, any comparison to previous collected data on hBM-MSCs is extremely difficult. Further hBM-MSCs are widely reported to express tenogenic markers by means of mechanical stimulation, such as cyclic strain [28,37,41]. Indeed, though hWJ-MSCs possess many properties of adult mesenchymal stem cells, they resemble pluripotent embryonic stem cells, and maybe would require a more complex environment to be committed toward a specific phenotype and could be not directly responsive to a specific mechanical cue.

When the second experimental set was organized with addition of nanocarriers for the controlled delivery of hGDF-5 within the 3D environment, a different cell behavior was observed with a more pronounced expression of tenogenic markers, including strong upregulation of the transcriptional factor SCX supported by a consistent overexpression of the other downstream genes, COL1A1 and DCN.

The Scleraxis gene encodes a basic helix-loop-helix (bHLH) transcription factor and is expressed in cells and progenitors of all tendon tissues; indeed, SCX −/− mice displayed severe tendon defects [60]. Moreover, SCX is essential in the fate determination of MSCs towards tenogenic differentiation in vitro, up-regulating other characteristic genes, such as COL1A1, DCN, and Tenomodulin (TNMD) [61]. Furthermore, it is well known that the collagen fibril structure of tendon ECM is determined and maintained by small leucine-rich proteoglycans (SLRPs), such as Decorin [62]. Moreover, if on the one hand Scleraxis is involved in tendon mechanoresponse [29,49,63], on the other type III collagen is inversely correlated to tendon modulus [64] or is expressed at the rupture sites of human tendons [65]. Therefore, it could be considered a negative marker during tendon healing and regeneration processes [13].

The qRT-PCR data are largely favorable with the use of nanocarriers as a drug delivery system within the 3D scaffold to ensure the controlled delivery of biomolecules that can act as specific inputs. The peptide release was not properly constant along the culture (see Figure 6d); however, the overall concentration assured was effective. The drug release by PLGA system is controlled by multiple events. First, the NCs undergo to a water wetting that allows the drug release by diffusion. This release is typical for the first days and promotes the so-called “burst effect” that involves the drug entrapped mainly on the biopolymer carrier surface. Meanwhile, the water diffusion within the biopolymer begins polymer hydrolysis, which is further promoted by the decreasing of the local pH values due to the acid monomer concentration. This hydrolysis reaction promotes bulk erosion of the polymer and then further drug release. These mechanisms compete and overlap over the release time assuring a quite linear drug release along the first 15 days [29,50,59,66]. Therefore, the release profile can display a nonlinear behavior, especially when an extremely low drug loading is adopted, as was likely the case here. Furthermore, due to mass transfer constraints related to the above-described events, nanocarriers use within a 3D environment should be avoided in static culture. Dynamic conditions ensure the proper convective mass transfer that is required to assure a proper drug release profile [32]. This aspect is extremely important and implies that even if a mechanical input does not always have a direct effect on cells, it can play a role in a cooperative action to ensure mass transfer and assure the correct release kinetics of the drug delivery nanodevices inserted within the 3D scaffold.

Given the well-known hWJ-MSCs immunomodulatory activity [9,67], gene expression of several pro-inflammatory and anti-inflammatory cytokines along the culture was explored in nanocarriers-supplemented culture that had shown the best tenogenic commitment. An inflammatory infiltrate with a high content of pro-inflammatory cytokines such as IL-6, TNF-α, and IL-17 has been identified in tendon biopsies during the initial phase of the tendinopathy process [68]. Inflammation is the first of the three main phases during the tendon healing process, followed by proliferation and remodeling. Each phase is influenced by a temporally and spatially controlled release of mediators by cells [69]. Our data suggested that hWJ-MSCs expressed key immunomodulatory molecules when undergoing tenogenic differentiation in vitro with a consequent potential ability to modulate the inflammatory response. The overexpression of pro-inflammatory factors (IL-6, TNF) was evident, together with upregulation of anti-inflammatory ones (IL-10, TGFβ1), probably suggesting an attempt by cells to support differentiation. In this sense, further investigations are required to better understand this finely tuned process. However, the described 3D system was confirmed to be an extremely interesting tool for the study of tendon regenerative events and the related drug activity when delivered by the nanocarriers assembled within the 3D system.

## 5. Conclusions

The present work described an innovative biomimetic 3D elastomeric construct with nano-functionalization and its in vitro evaluation for biosafety and improvement in teno-regenerative properties. The elastomeric 3D scaffold was assembled with biopolymer microspheres carrying hGDF-5, and mesenchymal stem cells isolated from Wharton’s Jelly. Compared with hGDF-5 supplemented culture medium, when PLGA/hGDF-5 were used in the 3D system, hWJ-MSCs showed increased tenogenic marker expression and collagen deposition within the fibrin matrix. We also hypothesized that the dynamic culture of the 3D system was important to assure convective mass transport of hGDF-5 plus provision of the adequate sink conditions required to provide a proper release profile.

Furthermore, despite the limited descriptions of hWJ-MSCs in tendon tissue-engineering protocols, our data suggested that hWJ-MSCs are useful and provide a potentially advantageous alternative for in vitro studies within regenerative protocols. An immunomodulatory activity, in relation to tenogenic commitment, was also observed, but it remains to be understood what the involvement of hWJ-MSCs is, as well as the role of bio-functionalized constructs in the stimulation of inflammatory reactions. Future evolution of the in vitro model described can be the controlled release of multiple growth factors with independent release kinetics in order to mimic complex patterns (spatial and temporal) of growth factor presentation to cells. The 3D system can be used as an advanced in vitro model for the study of controlled delivery formulation related to tendon regenerative events.

## 6. Patents

The SEE technology for nanocarriers fabrication was described in the US Patent US/8628802 B2 Jan 2014. Inventors: Reverchon E., Della Porta G., Continuous process for micro-spheres production by using expanded fluids. Applicant: University of Salerno.

## Figures and Tables

**Table 1:** Mechanical characterization of hyaluronate braided band and bioengineered 3D construct.

	Braided Band	3D Construct
Humidity (%)	100	100
Modulus of elasticity (MPa)	6	2
Elongation at break (%)	85	78
Tensile strength at break (MPa)	3	1

## Supplementary Materials

The following are available online at https://www.mdpi.com/article/10.3390/pharmaceutics13091448/s1, Figure S1: Flow cytometry characterization of hWJ-MSCs. Figure S2: The 3D system coupled with the customized bioreactor.
