# Supplementary material for: 3D Biomimetic Scaffold for Growth Factor Controlled Delivery: An In-Vitro Study of Tenogenic Events on Wharton’s Jelly Mesenchymal Stem Cells
Source: Pharmaceutics. 2021 Sep 10;13(9):1448. doi: 10.3390/pharmaceutics13091448 (PMC8465418; doi:10.3390/pharmaceutics13091448)
Supplement: Supplementary file 1 [file pharmaceutics-13-01448-s001.zip › pharmaceutics-1341568-supplementary.pdf]

Supplementary Materials: 3D Biomimetic Scaffold for Growth Factor Controlled Delivery: An In-Vitro Study of Tenogenic Events on Wharton's Jelly Mesenchymal Stem Cells

Maria Camilla Ciardulli, Joseph Lovecchio, Pasqualina Scala, Erwin Pavel Lamparelli, Tina Patricia Dale, Valentina Giudice, Emanuele Giordano, Carmine Selleri, Nicholas Robert Forsyth, Nicola Maffulli and Giovanna Della Porta

hWJ-MSCs isolation and harvesting

hWJ-MSCs were prepared from fresh human umbilical cord obtained during normal spontaneous vaginal delivery. Briefly, umbilical cord sections, approximately 7.5 cm long, were placed in 0.9% NaCl physiological solution supplemented with Ampicillin-Sulbactam 1 g + 500 mg, stored at 4°C, and processed within 4 h of collection. The umbilical cord was cut into 2.5 cm segments, and washed in fresh transport media to remove blood and debris. Each umbilical cord segment was sectioned longitudinally with sterile scissors, and the visible arteries and veins removed. Each piece was transferred to a tissue culture flask (175 cm^2) (BD Falcon, Bedford, MA, USA) containing α-MEM (Corning Cellgro, Manassas, VA, USA) supplemented with 10% FBS (Corning Cellgro, Manassas, VA, USA), 1% Glutagro™ (Corning Cellgro, Manassas, VA, USA), and 1% Penicillin-Streptomycin solution. Cultures were incubated at 37 °C in a humidified atmosphere containing 5% CO_2. Cell growth was monitored daily with changes of media twice a week. Upon reaching 100% confluence, cells were detached using 0.05% trypsin-0.53mM EDTA (Corning Cellgro, Manassas, VA, USA) and washed with PBS 1X (Corning Cellgro, Manassas, VA, USA), counted using Trypan Blue solution (Sigma-Aldrich, St. Louis, MO, USA), and sub-cultured at a concentration of 4×10^3 cells/cm^2. For hWJ-MSCs immunophenotype characterization flow cytometry analysis was performed on cells obtained at Passage 1.

Flow cytometry and gating strategy

hWJ-MSCs were detached and counted; 1×10^5 cells were incubated at RT for 20 min with the following directly conjugated mouse-anti human antibodies: CD90 FITC (Beckman Coulter, Fullerton, CA, USA), CD73 APC (Miltenyi Biotec, Gladbach, Germany), CD105 PE (Beckman Coulter, Fullerton, CA, USA), CD45 PC7 (Beckman Coulter, Fullerton, CA, USA), HLA class-II FITC (Beckman Coulter, Fullerton, CA, USA), CD14 PC7 (Beckman Coulter, Fullerton, CA, USA) and CD34 PE (Beckman Coulter, Fullerton, CA, USA). The isotype-matched immunoglobulins IgG1 FITC (Beckman Coulter, Fullerton, CA, USA), IgG1 PE (Beckman Coulter, Fullerton, CA, USA), IgG1 APC (Beckman Coulter, Fullerton, CA, USA), and IgG1 PC7 (Beckman Coulter, Fullerton, CA, USA) were used as negative controls under the same conditions. At least 15,000 total events were acquired with a BD FACSVerse flow cytometer (Becton Dickinson, BD, NJ, USA). Further analysis and plots were generated using the BD FACSuite analysis software. Statistics are summarized in Figure S1.

A minimum of 1×10^5 cells were stained for mesenchymal phenotype analysis. Manufacturer's instructions of used antibodies (Beckman Coulter) were optimized as follows. For antibody mix 1, 2.5 μL of fluorescein isothiocyanate (FITC) - conjugated anti-CD90, 10 μL of phycoerythrin (PE) - conjugated anti-CD105, 5 μL of allophycocyanin (AOC) – conjugated anti-CD73, and 10 μL of phycoerythrin cyanin 7 (PC7) – conjugated anti-CD45 antibodies were added. For antibody mix 2, 10 μL of FITC – conjugated anti-HLA-DR, 10 μL of PE – conjugated anti-CD34, and 10 μL of PC7 – conjugated anti-CD14 antibodies were added. After 20 min incubation at room temperature, samples were washed twice with phosphate buffered saline (PBS) and resuspended in the same buffer for acquisition.

FACSVerse cytometer (BD Biosciences) equipped with 2 lasers (blue, 488 nm, and red laser, 628 nm) and BD FACSuite software (BD Biosciences) were used for sample acquisition. Compensation was calculated using single-color controls for each fluorochrome and an unstained sample was used as negative control for setting PMT voltages. All samples were run using the same PMT voltages. A minimum of 30,000 events were recorded.

FlowJo software (v.10.7.1, LLC, BD Biosciences) was employed for post-acquisition compensation and flow cytometric analysis. After post-acquisition compensation using FlowJo, Wharton's jelly cells were first identified using linear parameters (forward scatter area [FSC-A] vs side scatter area [SSC-A], and double cells were excluded (FSC-A vs FSC-H). For antibody mix 1, CD90 and CD45 expression was investigated on single cells, and CD105 and CD73 expression was further studied on CD45-CD90+ cells. For antibody mix 2, HLA-DR and CD34 expression was first investigated on single cells, and CD14 was further studied on CD34-HLA-DR- cells. Expression of each marker on single cells was also reported using histograms and using unstained samples as negative controls.

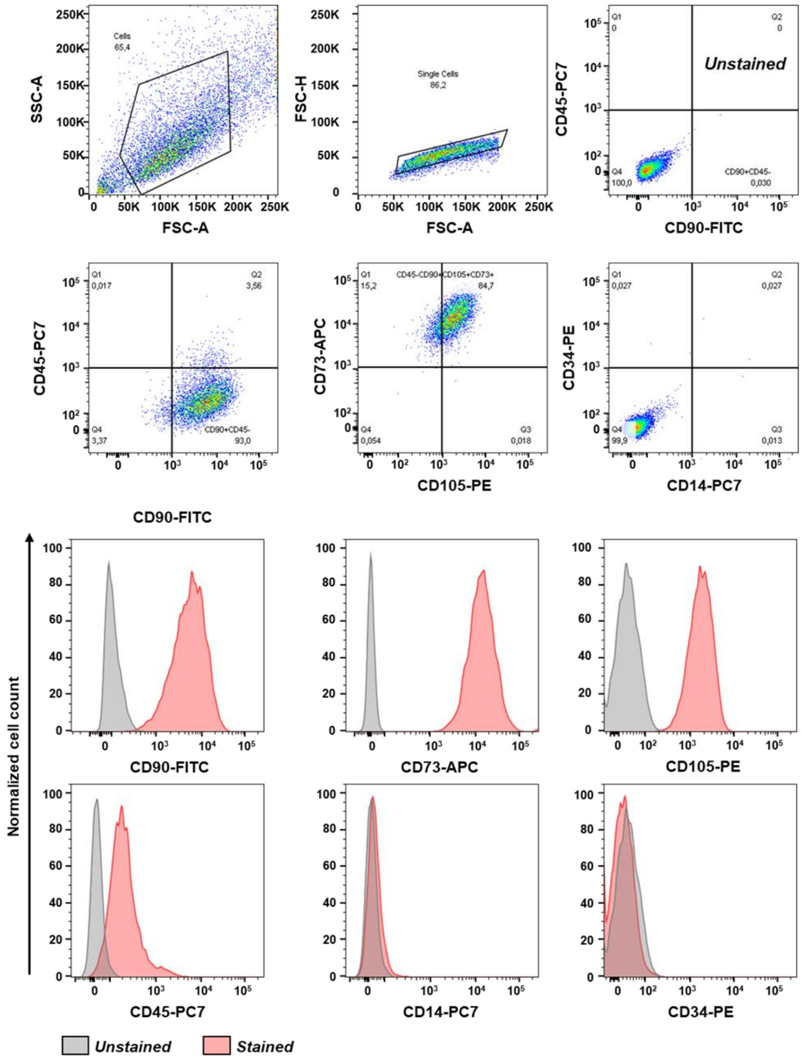

Figure S1. Flow cytometry characterization of hWJ-MSCs. The panel shows the representative flow cytometry events of forward scatter (FSC) vs. side scatter (SSC), excluding double cells (FSC-A vs FSC-H).

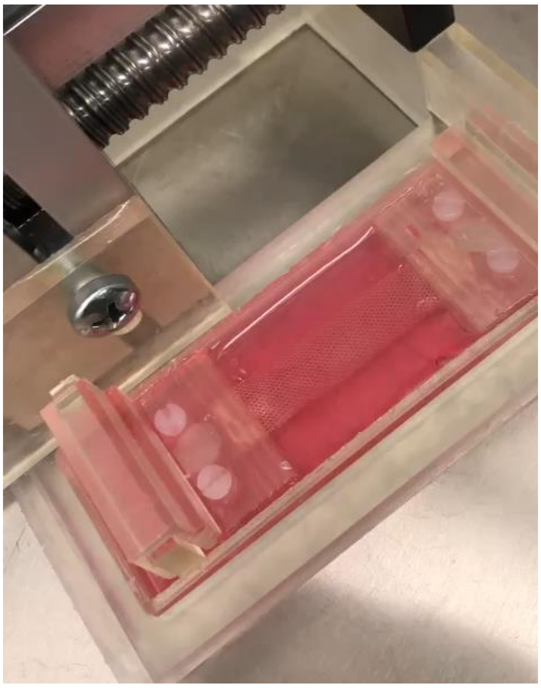

Figure S2. The 3D system was able to transmit a cyclic strain to the cells by mean of a hyaluronated braided band coupled with a customized bioreactor.
